# Management of the HBV reactivation in isolated HBcAb positive patients affected with Non Hodgkin Lymphoma

**DOI:** 10.1186/1471-230X-14-31

**Published:** 2014-02-17

**Authors:** Mario Masarone, Amalia De Renzo, Vincenzo La Mura, Ferdinando Carlo Sasso, Marco Romano, Giuseppe Signoriello, Valerio Rosato, Fabiana Perna, Fabrizio Pane, Marcello Persico

**Affiliations:** 1 Internal Medicine and Hepatology Unit, University of Salerno, Via Allende, Baronissi (Salerno) CAP: 84081, Italy; 2 Haematology Department, Federico II University of Naples, Naples, Italy; 3 AM Migliavacca Center for Liver Disease, First Division of Gastroenterology, Fondazione IRCCS Cà Granda Ospedale Maggiore Policlinico, Università degli Studi di Milano, Milan, Italy; 4 Internal Medicine and Hepatology Department, Second University of Naples, Naples, Italy; 5 Gastroenterology and Endoscopy Department, Second University of Naples, Naples, Italy; 6 Department of Statistical Sciences, Second University of Naples, Naples, Italy

**Keywords:** Occult HBV infection, Non Hodgkin Lymphoma, HBV reactivation, Immunosuppression

## Abstract

**Background:**

Occult HBV infection (OBI) is defined by the persistence of HBV in the liver without serum HBsAg and HBVDNA. It represents a life-threatening event during immunosuppressive chemotherapies. An OBI occurs in approximately 18% of HBcAb + patients. International guidelines suggest surveillance for HBV markers in immunosuppressed patients. In Non-Hodgkin Lymphoma (NHL), the prevalence of OBI reactivation remains to be established.

**Methods:**

In order to determine the prevalence of occult HBV reactivation in a large cohort of patients during chemotherapy for NHL, we analysed 498 NHL patients in a centre of Southern Italy. We evaluated HBV markers, NHL type, treatment type and occurrence of HBV reactivation.

**Results:**

Forty % of patients were treated with monoclonal antibodies and 60.3% without. Ninety-six patients were HBcAb+, HBsAg-. HBV reactivation occurred in ten subjects of this subgroup. All of them were successfully treated with Lamivudine. None of the patients experienced liver-related death. The prevalence of OBI reactivation was of 10.42% in HBcAb + HBsAb- patients. This event occurred in 50% of patients treated with mild immunosuppressive therapies. Each reactivation was treated with Lamivudine.

**Discussion:**

This report suggests that a strict surveillance is important and cost-effective in HBcAb + HBsAg- NHL patients treated with mild immunosuppressive therapies, in order to detect an occult HBV reactivation.

## Background

“Occult” HBV infection represents a particular clinical entity that is characterized by the persistence of HBV DNA in the liver tissue, without the evidence of overt HBV infection, in individuals who are HBsAg negative and HBcAb positive or negative [1]. It has also been episodically reported in HBsAb positive patients [2]. Its characteristics are: the absence of HBV DNA [or eventually transient presence of very low levels of viraemia] in the serum, and the persistence in the liver of the “covalently closed circular DNA” (cccDNA), a long-lasting HBV replication intermediate that can be revealed only by very sensitive techniques like “nested-PCR”, performed on liver tissue [1]. It is an “elusive” infection, the real prevalence of which in the general population is not known, being quite variable depending on the different geographical areas and study populations [3]. However, few studies report that the prevalence of OBI is approximately 16%-18% in subjects with evidences of previous HBV infection (i.e. HBcAb positive/HBsAg negative patients) and of 7-8% in subjects totally seronegative for HBV [4-6]. What is well known about this silent infection is that it can represent a life-threatening risk factor if the carrier experiences an immunosuppression. In fact, when the host immune surveillance is low, an overt HBV reactivation can occur [2]. In this case, the patient has titrable HBsAg and HBV DNA in the serum and, as soon as the immune surveillance is re-constituted at the end of chemotherapy, he develops an acute hepatitis that can range from simple lobular hepatitis with ALT elevation and only minimal lesions, to fulminant liver failure and death. Therefore, any patient who carries the OBI, and necessitates a chemotherapy-immunotherapy, should undergo to pre-emptive antiviral therapy with nucleoside/nucleotide analogues that have demonstrated to be efficacious in preventing HBV reactivation in various immunosuppressive settings [7-15]. The core of the problem is that OBI cannot be easily diagnosed and, for this reason, any HBcAb positive/HBsAg negative patient should be considered a possible occult infection carrier. What we know from literature is that onco-hematological diseases have the major risk of OBI reactivation, because of the strong immunosuppression experienced by the patients, due to both the disease itself and the chemotherapy [2]. In particular, in non-Hodgkin lymphoma (NHL), occult HBV reactivation has been reported to occur in 3% to 25% of patients, depending on the pharmacological and geographical settings [16-20]. Even if the real prevalence remains to be established, American Association for the Study of Liver Diseases (AASLD) recommended periodical monitoring of serum HBsAg and HBV-DNA, [21] whereas European Association for the Study of the Liver (EASL) recommended monitoring with serum ALT and eventually HBV-DNA assays in these patients [22]. The Italian association for the Study of the Liver (AISF) also published its recommendation in 2007 indicating two different strategies: for mild haematological therapies (standard protocols without monoclonal antibodies) HBsAg monitoring was advised, whereas in subjects treated with intense immunosuppression (i.e. protocols including monoclonal antibodies and/or strongly immunosuppressive therapies, i.e. “dose dense” regimens) universal prophylaxis was indicated. Nevertheless the strength of the recommendation was low (B and C) and derived from retrospective studies. Further studies where encouraged in HBcAb positive patients [23].

This single-center retrospective study was designed to determine the prevalence of occult HBV reactivation in HBsAg-negative and HBcAb-positive carriers who underwent immunosuppressive treatments for malignant lymphomas in a large cohort of patients from Southern Italy.

## Methods

From January 2005 to December 2011 we enrolled 498 consecutive patients admitted to the Haematology Division of Federico II University of Naples for treatment of Non-Hodgkin lymphoma.

Exclusion criteria were: the presence of any haematological malignancy other than Non-Hodgkin Lymphoma, previous immunosuppressive treatments of any kind (organ transplant, autoimmune therapies, other malignancies), HIV infection.

Each patient gave an informed consent, and the study was approved by the Ethics Committee of the University of Salerno. Non-Hodgkin lymphoma was diagnosed from histological findings (i.e. lymph nodes, bone marrow, etc.) according to the Revised European-American Lymphoma (REAL) classification criteria revised by Harris [24]. It was classified according to the REAL classification and grouped into indolent or aggressive lymphomas [25]. Ann Arbor classification stage was determined for all patients at the onset of the neoplastic disease by physical examination, total body computed tomography scan and bone marrow biopsy. Cheson’s criteria were used to define the response to antineoplastic treatment [26].

Prior to start NHL treatment every single patient underwent a physical examination, complete blood count, ALT and AST, routine biochemistry assays, HBsAg, HBcAb, HBeAg, HBeAb, HBsAb, HAVAb IgM (antibodies by ELISA Orthodiagnostic system), HCV-Ab (with commercial enzyme linked immunosorbent assay III, Abbot laboratories Chicago). HBV-DNA with Real-Time PCR (LightCycler Instrument, Roche Molecular Biochemicals, Mannheim, Germany), was also performed in patients who were already HBsAg positive patients before NHL treatment.

**Figure 1:**
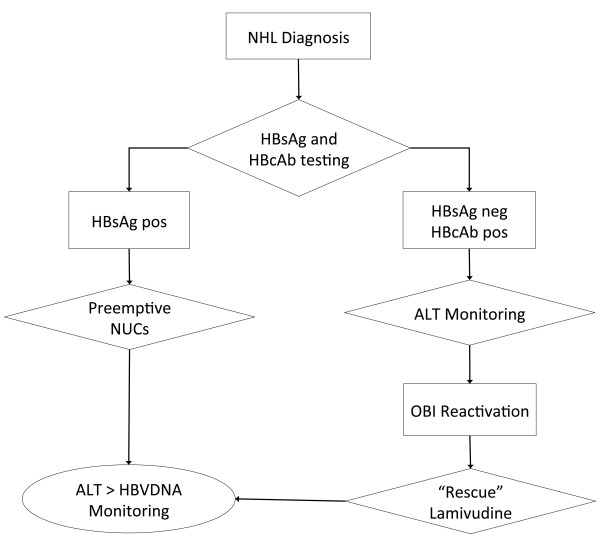
**Diagnostic/therapeutic flow-chart.** Every patients underwent to HBV status assessment prior to start chemotherapy. HBsAg positive patients underwent to NUCs pre-emptive therapy. Monitoring of ALT continued monthly for every patient during treatment and at least 16 months after treatment interruption. If there were an ALT elevation, HBV-DNA and HBsAg assays were performed to diagnose probable OBI reactivation. At the diagnosis of probable OBI reactivation a “rescue therapy” with lamivudine was promptly started. In patients who reactivated, ALT every two weeks and a monthly complete liver functionality test plus HBV-DNA assay were performed. In patients who reactivated, Lamivudine was continued until the complete HBV remission (see diagnostic criteria in the Methods section).

All HBsAg positive patients were evaluated by a skilled hepatologist who assessed the liver disease status and performed the correct follow-up of the liver functionality. Every patient who was HBsAg positive underwent to chemotherapy after a pre-emptive therapy with NUCs was assured, as indicated by international guidelines. Pre-emptive therapy started at least in a time interval between two weeks before and the first day of chemotherapy (depending on the urgency to initiate NHL treatment), and continued throughout the whole time of chemotherapy, and at least 12 months later. An HBV-DNA assay was performed every 2 months during treatment and 16 months later. Every HBcAb positive, HBsAg/HBsAb negative patient underwent to monthly ALT monitoring during therapy and throughout 16 months after. All the others patients underwent to a monthly ALT monitoring during therapy and at least every 3 months in the follow up. Thereafter every patient underwent to twice a year liver ALT evaluation. If the patient experienced an ALT derangement more than twice upper normal value (UNV), complete blood tests were performed to search liver-bound viruses: HBV-DNA, EBV-DNA, HCV-RNA, CMV-DNA and HSV-RNA 1 and 2 assays were investigated. If the patient experienced a probable OBI reactivation (see the diagnostic criteria below), Lamivudine therapy was promptly started at the standard dosage. At OBI reactivation ALT monitoring was performed every two weeks and complete liver functionality tests (comprehending also HBV-DNA quantitative assay) were performed monthly. The study protocol is shown in Figure 1.

### Definition of HBV/OBI reactivation

An “HBV reactivation” was diagnosed when a patient experienced an ALT/AST derangement (at least 2x upper normal values) with HBsAg and HBV-DNA detectable in the serum (>2000 UI/mL), without any contemporary possible cause of liver disease. A “probable OBI reactivation” diagnosis was performed when diagnostic criteria above exposed were applicable to a patient who was HBsAg negative before NHL treatment (reverse seroconversion), and any other cause of liver functionality derangements was excluded as described above. In these patients a HBV DNA assay was performed on blood samples collected and frozen prior to start treatment, at the time of enrolment.

### Cost effectiveness analysis

A cost effectiveness analysis was performed to evaluate the more effective approach between universal prophylaxis and monitoring to prevent OBI reactivation risk in HBcAb positive patients undergone to Rituximab containing protocols. We used the “Diagnosis Related Group system” (DRG), that is used to assess the healthcare costs in our country [27].

### Statistical analysis

Statistical analyses were performed using the Statistical Program for Social Sciences (SPSS®) ver.16.0 for Macintosh®(SPSS Inc., Chicago, Illinois, USA). Student t-test and Mann-Whitney U test were performed to compare continuous variables, chi-square with Yates correction or Fisher-exact test to compare categorical variables. Statistical significance was defined when “*p < 0,05*” in a “two-tailed” test with a 95% Confidence Interval.

## Results and discussion

Demographical characteristics of our study population is shown in Table 1. Of the 498 patients, 45,4% were females and 54,6% males, with a mean age of 61 years (SD ± 14). 52,7% of these patients had an aggressive lymphoma and 47,3% an indolent lymphoma according to Percy et al. [22]. Four hundred and 76 (95,58%) patients had B-cell lymphoma, 22 (4,41%) patients had T-cell lymphoma. Thirty-eight pts (7,6%) were HBsAg positive. Of these patients, 6 (15,78%) were inactive carriers (HBsAg positive with HBVDNA < 2000 UI/mL and normal ALT), 32 (84,22%) had an HBV active disease, no one had decompensated cirrhosis. Moreover, 15 (39,47%) were already in treatment with nucleoside analogues (5 with LAM + ADF, 5 with ETV, 5 with TFV), 17 where considered to have mild liver disease and were not under antiviral treatment at the time of NHL diagnosis. HBsAg positive patients not already in treatment with NUCs, were posed in pre-emptive antiviral therapy with nucleoside analogues. Ninety-six out of 498 patients (19,27%) were HBcAb positive and HBsAg negative. This prevalence is higher prevalence in respect to the general population, but has already been described in NHL patients [28]. No statistical relevant differences were found between HBsAg positive and HBsAg negative patients except for the HBV reactivations that occurred only in HBsAg negative patients, as described above. Ninety (18,07%) patients were HBsAb positive, and underwent to the same monitoring as the totally HBV negative ones. Of these, 30 subjects were HBsAb and HBcAb positive, and therefore were likely to have spontaneously recovered from an HBV infection. The remaining 60 subjects were HBsAb positive only, and therefore likely vaccinated.

**Table 1:** Demographical characteristics of the study population

**Variable**	**Overall**	**HBsAg positive**	**HBsAg negative**	***p***
No. of patients	498	38	460	*-*
Age - Mean [±SD]	61,00 [±14,05]	55,42 [±13,62]	57,32 [±14,95]	*0,45*
Sex – M/F	M: 54,40% -	M: 57,89%	M: 47,83%	*0,23*
	F: 45,40%	F: 42,11%	F: 52,17%	
No. of patients HBsAb +	90 [18,07%]	0	90 [19,56%]	*0,003*
No. of patients HBcAb +/ HBsAg -	134 [26,90%]	0	96 [20,86%]	*0,002*
No. of patients HBcAb - / HBsAg -	274 [55,03%]	0	274 [59,56%]	
Lymphoma type	Indolent: 47,3% -	Indolent: 33,35% -	Indolent: 44,34% -	*0,22*
	Aggressive: 52,7%	Aggressive: 66,65%	Aggressive: 55,66%	*0,22*
Rituximab therapy	Yes: 60,30% -	Yes: 18 [47,37%]	Yes 262 [56,95%]	*0,25*
	No: 39,70%	No: 20 [52,63%]	No: 198 [43,04%]	
HBV reactivation	10 [2,01%]	0	10 [2,17%]	*0,22*
NUC profilaxys/rescue therapy	38/10	38/0	0/10	*<0,0001*
“Per year Incidence of reactivation” [mean ± SD]	1,71%	0	2,04%	*<0,0001*
	±2,43%		±2,97	
Liver related decompensations/hospitalizations/deaths	0%	0%	0%	*-*

In the first column overall patients characteristics: HBV reactivation occurred in 2,01% of cases. In the second and third column demographical characteristics of HBsAg positive and negative patients, respectively. HBV reactivation occurred in HBsAg negative patients only, due to the NUC profilaxys performed in HBsAg positive patients [see Methods]. (Chi-square, Pearson corrected calculations for all variables, Age: student t-test).

### Therapy type

On the basis of the clinical assessment of the lymphoma, 39,7% of the patients underwent anti-neoplastic treatments protocols that included rituximab or high immunosuppressive drugs or dosages, 60,3% followed conventional treatment protocols not including highly immunosuppressive drugs or “dose dense” regimens [23]. Due to the retrospective nature of the present study the prevalence of Rituximab-containing protocols is slightly lower than that observed in the actual clinical practice. In HBcAb positive-HBsAg negative patients NHL chemotherapy protocols included Rituximab in 50%. Table 2 reports on this class of patients.

**Table 2:** HBcAb positive “only” patients characteristics

		**Rituximab containing protocols**		
**Variable**	**Overall**	**Yes**	**No**	***p***
No. of patients	96	48	48	*ns*
Age - Mean [±SD]	64,39 ± 9,63	65,17 ± 10,21	64,02 ± 8,37	*ns*
Sex – M/F	M: 57	M: 30	M: 27	*ns*
	F: 39	F: 18	F: 21	
Lymphoma type	Indolent: 50	Indolent: 17	Indolent: 33	*0,001*
	Aggressive: 46	Aggressive: 31	Aggressive: 15	
Probable OBI reactivation	10 [10,42%]	5 [10,42%]	5 [10,42%]	*ns*
NUC profilaxys/rescue therapy	0/10	0/5	0/5	*ns*
“Per year Incidence of reactivation” [mean ± SD]	8,23% ± 9,84	9,08% ± 10,69	11,54% ± 40,74	*ns*
Reactivation time [mean weeks after therapy]	26,67 ± 12,20	31,60 ± 14,50	20,50 ± 4,72	*ns*
Liver related decompensations/hospitalizations/deaths	0%	0%	0%	*-*

No statistical differences were found in patients treated with ad without Rituximab. (Chi-square, Pearson corrected calculations for all variables, Age, incidence of reactivation and reactviation time: student t-test).

No statistical significant differences were found between patients treated with and without Rituximab in this subgroup, except for the different rates of aggressive lymphomas that were more frequently treated with Rituximab.

### HBV reactivation/OBI reactivation

A mild ALT (mean 113,44 ± 61,35 U/dL) derangement was found during treatment in 47 patients (9,44%) without evidence of any liver infection and this was attributed to an effect of chemotherapy.

No OBI reactivations were diagnosed in HBsAb positive, in HCVAb positive and totally negative patients.

A probable OBI reactivation was observed in 10 patients out of 96 HBcAb positive-HBsAg negative patients (10,42%), who had clinical and laboratory characteristics compatible with HBV reactivation criteria described above (see Methods section). Characteristics of the 10 reactivated patients are summarized in Table 3.

**Table 3:** Characteristicsof “HBVreactivated” patients. 10 patients over 498 had HBV reactivation

**Patient no.**	**Age (years)**	**Sex**	**Stage of lymphoma**	**Indolent aggressive (0-1)**	**Baseline**					**At diagnosis of HBV reactivation**		**Time of diagnosis of HBV Reactivation**	**Time to HBV recovery**	**Nuc therapy duration**	**Follow up HBsAg status**	**Outcome**
					**HBs-Ag**	**HBV- DNA (UI/μL)**	**ALT (U/L)**	**Rituximab (yes/no)**	**Therapy**	**HBV- DNA (UI/μL)**	**Peak ALT (XN.V.)**	**Weeks after NHL therapy**	**Weeks after NUC start**	**Weeks**	**Pos/neg**	
1	50	F	IV	1	-	0	23	y	CHOP-R	350.000	4,5x	40	4	28	Neg	Alive (NHL remission)
2	73	M	IV	0	-	0	26	n	VNCOP-B	450.000	10x	24	8	Ongoing	Neg	Alive (NHL relapse)
3	64	F	I	1	-	0	34	y	Fludara-R	650.000	12x	12	4	22	Neg	Alive (NHL remission)
4	62	M		1	-	0	22	y	CHOP-R	130.000	4x	44	3	Ongoing	Pos	Alive (NHL relapse)
5	50	F	II	1	-	0	28	y	CHOP-R	910.000	20x	20	8	44	Neg	Alive (NHL remission)
6	71	M	IV	0	-	0	19	n	CHOP	160.000	11x	24	6	48	Neg	NHL related death
7	66	M		0	-	0	36	n	VNCOP-B	170.000	3,7x	20		Ongoing	Pos	Alive
8	70	M	IV	0	-	0	31	y	Fludara Nova -R	160.000	5,4x	72		Ongoing	Pos	Alive
9	52	F	I	0	-	0	29	n	CHOP	550.000	6,1x	14	7	46	Neg	Alive
10	60	M	IV	0	-	0	34	n	CEOP, Gem, VNCOP-B	340.000	6x			Until death	Pos	NHL related death

Of this group, everyone had HBcAb positive, HBsAg, HBsAb and HBVDNA negative status prior to start NHL therapy. Five patients were treated with Rituximab-containing protocols and five without. The mean time to HBV reactivation was 26,67 (±12,21) weeks. The mean time to HBV recovery (HBVDNA negativization and normal ALT) was 5,71 (±2,06) weeks. Five patients had HBsAg negativization after a mean time of NUC therapy of 37,80 (±11,78) weeks and stopped NUC therapy. Five patients were already in NUC therapy at the end of follow-up, due to HBV infection persistence (HBsAg positivity) and/or NHL relapse. Patient 7 to 10 have missing data, because of the retrospective nature of this study.

Of the reactivated patients (6 M, 4 F, mean age 63 y, SD ± 8), 2 had T–cell NHL and 8 B NHL (3 MALT, 5 Diffuse Large B cells). Of these subjects, 5 (5,21%) had been treated with Rituximab (3 with CHOP-R protocol, 1 with Fludarabin and Rituximab, 1 with Fludarabin + Novantrone + Rituximab) and 5 (5,21%) without (2 with Fludarabin, 1 with CHOP, 1 with VNCOP-B, 1 with CEOP). Each patient experienced reactivation after the end of chemotherapy cycle then, as expected, after the immune response restoration. The mean time of the appearance of HBV reactivation was 26,67 (±12,21) weeks.

### Reactivation risk

In the overall population, the Relative Risk (RR) of developing a HBV reactivation by the use of Rituximab-containing protocols was of 1.540 (95% C.I.: 0.481-4.930) in respect of standard chemotherapies (p:0.525). In HBcAb + HBsAg- patients, RR was equal to 1.000, since reactivation occurred with the same frequency in patients treated with and without Rituximab.

To adequately address the risk of a pOBI reactivation in our 96 patients HBcAb + HBsAg, we carried out a multivariate analysis (performed by a binary logistic regression model) to analyse the factors that could affect its appearance. In particular, we analysed: age, sex, lymphoma type, grading, staging and the use of Rituximab-containing protocols as independent variables and pOBI reactivation as dependent variable. None of the analysed factors reached statistical significance as an independent factor influencing the occurrence of reactivation (see Additional file 1).

### Outcome and follow-up

Every patient who reactivated HBV infection was rapidly treated with nucleoside analogue Lamivudine experiencing a fast viral response with HBVDNA negativization with a mean time of 5,72 (±2,05) weeks. None had liver decompensation symptoms and signs, nor did any of them need hospitalization. In the follow-up, 2 patients died for NHL complications, 3 patients had NHL remission, 2 patients had NHL relapse and were treated again, 5 patients are already in follow-up. None experienced liver-related death in this group.

Because of the small number of patients who reactivated infection, we cannot make a statistically valid comparison with patients who did not reactivated, in terms of NHL-related mortality and morbidity. Moreover, due to the fact that we included various histological types of NHL in the present study, report data on overall mortality and morbidity may be methodologically wrong, and to report on mortality rate of every histological type on NHL falls out of the aims of the present study.

### Cost-effectiveness analysis

As reported above, according to AISF indications, HBcAb positive/HBsAg negative patients treated with Rituximab and/or “dose dense” regimens should undergo to universal prophylactic antiviral therapy with NUCs during NHL treatment [22]. In our series, the recurrence rate of HBV reactivation in HBcAb positive subjects was found to be 10,42%, and this event wasn’t related to an increase in mortality rate. For this reason our cost benefit analysis, comparing the potential costs of Lamivudine prophylaxis in 48 HBcAb positive HBsAg negative patients treated with rituximab, and the costs of an eventual HBV reactivation in terms of hospitalization costs, reported an advantage in the “monitoring” approach that was used in our patients in respect to universal prophylaxis. Data are reported in Table 4.

**Table 4:** Cost-benefit analysis of prophylaxis in respect to HBV reactivation by “Diagnosis Related Group” system in our 48 HBcAb positive HBsAg negative patients treated with Rituximab

	**Unitary cost**	**n. patients**	**Total per patient**	**Duration [days]**	**Total**
*Cost of prophylaxis*					
Lamivudine	€ 3,18	48	€ 152,64	360	€ 54.950,40
HBV DNA monitoring	€ 130,00	48	€ 6.240,00	6	€ 37.440,00
HBsAg monitoring	€ 17,00	48	€ 816,00	6	€ 4.896,00
AST/ALT monitoring	€ 5,74	48	€ 275,52	12	€ 3.306,24
*Total*	€ 155,92	48	€ 7.484,16		€ 100.592,64
*Cost of HBV Reactivation*					
HBV DNA monitoring	€ 130,00	48	€ 6.240,00	6	€ 37.440,00
AST/ALT monitoring	€ 5,74	48	€ 275,52	12	€ 3.306,24
Cost of DRG 205 [v24 Grouper]	€ 3.769,10	5	-	-	€ 18.845,50
*Total*	€ 3.904,84	-	-	-	€ 40.746,24

Drg 205: Liver disease except malignancies, cirrhosis, alcoholic hepatitis with cirrhosis.

Based on these data, we compared HBcAb positive patients who reactivated infection with those who did not, to search for any event predictors, so to obtain a staging of the risk of reactivation. None of the analysed characteristics, such as age, sex, the type and the degree of lymphoma, the type and duration of chemotherapy, proved to be statistically different in the two groups, probably also due to the small number of patients reactivated which reduced the power of statistical tests.

### Discussion

Our study reports a prevalence of “probable OBI reactivation” in NHL patients. It has to be noticed that this prevalence is similar to the supposed prevalence of OBI infection in blood donors in the same area [29-44]. This finding may confirm the general conviction that onco-haematological patients are at high risk of HBV reactivation if they are OBI carriers. We reported about a “probable OBI reactivation” because in in this large retrospective study we did not collect liver specimens, and consequently we cannot confirm the presence in the liver extracts of almost two out of three HBVgenes (core, X, surface) in patients who reactivated infection, which has been defined as the diagnostic criterion of OBI [1]. Nevertheless, we can safely assign the HBV events occurred in our patients to an OBI infection reactivation, because of the finding of HBVDNA negativity at the time of the NHL diagnosis. HBVDNA assays were performed with very sensitive methods such as CobasTaqman PCR, and for this reason we can exclude that our patients were “false occult” HBV infected patients, as described by Launay et al. [44]. Moreover, we previously reported that in a small group of HBcAb positive patients with available liver biopsies specimens, OBI was found in tissue specimens only in patients later experiencing a HBV reactivation after chemotherapy [7].

The general agreement of International Liver Associations about the management of this type of patients is well known [21-23]. In particular, even if a very careful monitoring is suggested, different approaches are reported, in particular regarding the type of anti-neoplastic therapy. In fact, it has been reported that a slightly higher prevalence of OBI occurs in patients treated with highly immunosuppressive treatments, in particular monoclonal antibodies such as anti-CD20 antibody Rituximab [2]. Because of these findings, Italian Liver Association suggested prophylaxis with Lamivudine in all HBcAb positive HBsAg negative patients who are assigned to highly immunosuppressive treatments for haematological malignancies [23]. This approach should be justified by the low toxicity of orally administered Lamivudine. Nevertheless, in our HBcAb-positive cohort, OBI reactivation occurred in patients treated with Rituximab and also in patients in whom it was not administered, with a prevalence higher than that reported in other series. This may be due to the different study populations and/or different therapy protocols. In fact, it has to be noticed that two out of these 10 patients were treated with Fludarabin that is accounted for high immunosuppression. For this reason, even if the therapeutic approach in these two patients was not based on a “dose-dense” protocol, it is likely that these two patients experienced at least a “moderate” immunosuppression, intermediate between that of Rituximab and standard protocols. Our approach of strict monitoring in all these patients was chosen in a time (year 2005) when there were less indications from literature than today. Nevertheless, none had serious consequences by this type of management. In fact, no patient had liver decompensation, liver related hospitalizations or liver-related death. As reported in the results section, 39,7% of our HBcAb positive HBsAg negative patients were treated with Rituximab-including or dose dense protocols. In this way, we should have used Lamivudine as prophylactic treatment in all of them to prevent 5 events, which were safely managed without consequences. Moreover, OBI reactivation occurred also in patients undergone to “standard” treatments. If we refer to these data, universal prophylaxis should have been applied to all HBcAb positive HBsAg negative patient. At this point, a cost-benefit issue arises: is it more cost-effective to treat all the HBcAb positive HBsAg negative patients with NUCs to prevent the OBI reactivation occurrence in a small quote of them, or may it be more effective a “wait and see” protocol? To assess this point, we performed a cost-effectiveness analysis whose results are reported in Table 4. We calculated the costs of prophylaxis in a time interval of twelve months, which encompasses the time of a standard Rituximab-containing chemotherapeutic protocol and a minimum time of follow-up. It has to be noticed that very often NHL patients need more than one therapy cycle to obtain NHL remission, and sometimes, if they do not obtain a complete remission, undergo to long-term, low-dose, “maintenance” therapies with Rituximab. These patients are at high risk of HBV reactivations due to the long times of immunosuppression. Nevertheless, even if our calculations underestimated the costs of prophylaxis, the “monitoring approach” resulted cost-effective. Moreover, patients treated with standard protocols should have been excluded from the eventual prophylaxis protocol. Nevertheless, even if in our series HBV reactivation did not lead to an increase in mortality/morbidity, this event still remains a life-threatening risk for the patients. Moreover, even though in our series no serious events in terms of morbidity and/or mortality occurred, in other literature reports a monitoring approach did not guarantee patients survival [16,45-49]. These detrimental results could be ascribed to the delayed start of NUC therapy if the monitoring is not adequately strict(less than one evaluation/month). Also, it has been reported that performing only the transaminase evaluation should not be acceptable to prevent severe reactivations [2]. Our monitoring approach resulted efficacious probably because of the monthly ALT assay was strictly observed.

Due to the retrospective nature of our study, we cannot draw any firm conclusion on which should be the best approach for patients with isolated HBcAb positivity who should undergo chemotherapeutic regimens (universal prophylaxis vs monthly ALT monitoring). A randomized controlled trial might be needed to properly address this issue.

## Discussion

Out of four-hundred-ninety eight NHL patients, HBV reactivation occurred only in HBcAb+, HBsAg-, HBsAb- subjects. None of the HBsAg + patients experienced such a reactivation since all of them were treated with pre-emptive Lamivudine therapy.

The rate of reported reactivation was similar either in patients treated with low immunosuppressive therapy or in those treated with high immunosuppressive therapy (Rituximab).

A “monitoring” approach, being more cost-effective than universal prophylaxis, should be chosen, either in low or high immunosuppressive treated subjects and a “rescue” antiviral treatment is advised in the case of reactivation occurrence. Nevertheless, the reported failure of rescue therapy [16,45-49] needs to be considered and might represent a limitation.

The main limitation of the study is its retrospective nature. A prospective randomized double bind trial is warranted and, at present, the universal prophylaxis should be considered the only possible approach.

Our study confirms the need of at least a strict surveillance of these patients in order to prevent OBI reactivations, which is a life-threatening condition if not rapidly recognized and treated.

## Abbreviations

DRG: Disease Related Grouping system; LAM: Lamivudine; NHL: Non-Hodgkin Lymphoma; NUCs: Nucleoside analogues; OBI: Occult HBV infection.

## Competing interests

None of the authors had a personal or financial conflict of interest.

## Authors’ contributions

MM participated to study conception and design, data analysis and interpretation, article drafting and revising it critically for important intellectual content, and gave final approval for publication. He also collected the data. ADR participated to study conception and design, data analysis and interpretation, article revising for important intellectual content, and gave final approval for publication. VLM participated to study conception and design, data analysis and interpretation, article drafting and revising it critically for important intellectual content, and gave final approval for publication. FCS participated to study conception and design, data analysis and interpretation, article revising for important intellectual content, and gave final approval for publication. MR participated to study conception and design, data analysis and interpretation, article revising for important intellectual content, and gave final approval for publication. GS participated to study conception and design, data analysis and interpretation, article revising for important intellectual content, and gave final approval for publication. VR participated to study conception and design, data analysis and interpretation, article revising for important intellectual content, and gave final approval for publication. He also collected the data. FP participated to study conception and design, data analysis and interpretation, article revising for important intellectual content, and gave final approval for publication. FP participated to study conception and design, data analysis and interpretation, article revising for important intellectual content, and gave final approval for publication. MP participated to study conception and design, data analysis and interpretation, article drafting and revising for important intellectual content, and gave final approval for publication. He is responsible for the overall content as guarantor. All authors read and approved the final manuscript.

## Pre-publication history

The pre-publication history for this paper can be accessed here:

http://www.biomedcentral.com/1471-230X/14/31/prepub

## Supplementary Material

Additional file 1 **Supplemental material: binary logistic regression analysis vs risk of reactivation in HBcAb + HBsAg- negative patients.** Age, sex, lymphoma type, grading, staging and the use of Rituximab-containing protocols were used as independent variables and pOBI reactivation as dependent variable. None of the analysed factors reached statistical significance as an independent factor influencing the occurrence of reactivation. Click here for file

## Fundings

This research didn’t received specific funds.
