# Supplementary material for: Management of the HBV reactivation in isolated HBcAb positive patients affected with Non Hodgkin Lymphoma
Source: BMC Gastroenterol. 2014 Feb 17;14:31. doi: 10.1186/1471-230X-14-31 (PMC3938973; doi:10.1186/1471-230X-14-31)
Supplement: Additional file 1 — Supplemental material: binary logistic regression analysis vs risk of reactivation in HBcAb + HBsAg- negative patients. Age, sex, lymphoma type, grading, staging and the use of Rituximab-containing protocols were used as independent variables and pOBI reactivation as dependent variable. None of the analysed factors reached statistical significance as an independent factor influencing the occurrence of reactivation. [file 1471-230X-14-31-S1.rtf]

	B	S.E.	Wald	df	Sig. (p)	OR	95% C.I.for OR	
							Lower	Upper	
Sex	0,200	1,137	0,031	1	0,861	1,221	0,132	11,336	
Age	-0,088	0,064	1,891	1	0,169	0,916	0,808	1,038	
Histological type	-0,068	0,191	0,127	1	0,722	0,934	0,643	1,358	
Lymphoma Grading (indolent/aggressive)	-0,193	1,447	0,018	1	0,894	0,824	0,048	14,045	
Staging	0,375	0,668	0,315	1	0,574	1,455	0,393	5,386	
Rituximab	0,473	1,357	0,122	1	0,727	1,605	0,112	22,934	
Constant	3,297	5,595	0,347	1	0,556	27,036			

Supplemental table: binary logistic regression vs OBI reactivation in HBcAb positive HBsAg negative patients
